# Evaluating Maternal Risk Factors Impacting Fetal Intima–Media Thickness of the Abdominal Aorta Measured at 28 Weeks of Gestation

**DOI:** 10.3390/jcm13216519

**Published:** 2024-10-30

**Authors:** Biliana Belovan, Zoran Laurentiu Popa, Adrian Ratiu, Cosmin Citu, Ioana Mihaela Citu, Ioan Sas

**Affiliations:** 1 Doctoral School, “Victor Babes” University of Medicine and Pharmacy, Eftimie Murgu Square 2, 300041 Timisoara, Romania; biliana.belovan@umft.ro; 2 Department of Obstetrics and Gynecology, “Victor Babes” University of Medicine and Pharmacy, Eftimie Murgu Square 2, 300041 Timisoara, Romania; popa.zoran@umft.ro (Z.L.P.); citu.ioan@umft.ro (C.C.); sas.ioan@umft.ro (I.S.); 3 Department of Internal Medicine I, “Victor Babes” University of Medicine and Pharmacy, Eftimie Murgu Square 2, 300041 Timisoara, Romania; citu.ioana@umft.ro

**Keywords:** fetal health, obstetrics, maternal–fetal medicine, risk factors

## Abstract

**Background and Objectives:** Cardiovascular disease risk can exist in utero, influenced by maternal health factors. This study evaluates maternal characteristics and biochemical markers that correlate with the fetal intima–media thickness (IMT), aiming to identify interventions that could minimize prenatal influences on later cardiovascular disease. **Methods***:* In this observational study approved by the Institutional Review Board at The Obstetrics and Gynecology Clinic of the Timisoara Municipal Emergency Hospital, we recruited pregnant women aged 15–40 years, divided into groups based on their lipid profiles and gestational diabetes risk. The data collection had, as its main focus, ultrasound measurements, along with demographic, clinical, and biochemical parameters. The IMT of the fetal abdominal aorta was measured at 28 weeks of gestation. **Results***:* Notable differences were observed in the TNF-alpha levels (8.66 ± 3.87 pg/mL vs. 4.96 ± 3.37 pg/mL), hsCRP levels (0.94 ± 0.46 mg/L vs. 0.60 ± 0.52 mg/L), and the area under the curve (AUC) for hsCRP at 0.738 with a sensitivity of 84.41% and specificity of 79.01%. Compound score 2, integrating inflammatory markers and lipid profiles, exhibited a good diagnostic accuracy (AUC = 0.789) with a sensitivity of 86.35% and specificity of 81.42%. A regression analysis indicated strong associations of TNF-alpha and hsCRP with an increased fetal IMT, suggesting potential early markers of cardiovascular risk, presenting hazard ratios (HRs) of 2.21 (95% CI: 1.15–5.28) and 2.87 (95% CI: 1.11–4.23), respectively, both with *p*-values of less than 0.0001. Compound score 2 further indicated an increased risk (HR = 4.27; 95% CI: 1.19–8.32). **Conclusions***:* Statistically significant correlations were found between an increased fetal IMT and elevated maternal inflammatory markers (TNF-alpha and hsCRP), suggesting that these could serve as early indicators of cardiovascular risk. This study supports the potential for targeted prenatal interventions to reduce cardiovascular risk factors from the fetal stage, emphasizing the importance of monitoring inflammatory markers in pregnant women at risk.

## 1. Introduction

Studies have shown that certain maternal conditions, such as dyslipidemia and metabolic inflammation, are linked to the structural and functional changes in the fetal cardiovascular system [1,2,3]. Understanding these relationships is critical, especially as cardiovascular diseases remain a leading cause of global morbidity and mortality, and can lead to multiple conditions in later life and adulthood that can expand the cardiovascular system [4,5,6]. Recent advancements in fetal imaging have facilitated the measurement of the intima–media thickness (IMT) of the abdominal aorta in utero, a method that offers a non-invasive marker of early atherosclerotic disease [7,8]. While an elevated fetal IMT could potentially indicate an increased cardiovascular risk, the direct association with adverse outcomes in later life remains under investigation [9]. Reports indicate that variations in the fetal IMT can begin as early as the second and third trimesters, suggesting that fetal exposure to adverse maternal metabolic environments and chemicals may initiate vascular remodeling early in life and additional unknown complications that can occur later in life [6,10,11].

Epidemiological data underscore the prevalence of maternal risk factors that may contribute to an altered fetal IMT [12]. For instance, maternal obesity, a condition affecting approximately 20% of pregnant women in developed countries, might be correlated with a thicker IMT during fetal life, indicating a potential transgenerational transmission of cardiovascular risk [13,14]. Furthermore, the incidence of gestational diabetes mellitus has been rising, currently affecting up to 10% of pregnancies in the United States alone. This trend is concerning given the established links between gestational DM and various fetal abnormalities, including those related to the cardiovascular system [15,16,17].

The role of maternal dyslipidemia in influencing the fetal IMT is also gaining attention. Some research evaluating mother–child pairs demonstrated that elevated maternal cholesterol levels during pregnancy are associated with a worsening fetal lipid profile and increased fetal IMT [18,19]. This association was particularly strong when the mothers exhibited hypercholesterolemia during the third trimester, highlighting critical periods during pregnancy when the maternal lipid levels could have the most profound impact on the fetal vascular structure.

Moreover, systemic metabolic inflammation during pregnancy, a condition often exacerbated by obesity and metabolic syndrome, presents another layer of risk to the developing fetal cardiovascular system [20]. While the specific pathways through which this inflammation impacts the fetal IMT are still being elucidated, the general consensus is that inflammatory conditions during pregnancy create a hostile uterine environment that can initiate or exacerbate vascular changes in the fetus.

By investigating these factors and their impact on the fetal IMT, researchers aim to develop targeted interventions and preventative measures that could significantly alter the trajectory of cardiovascular disease starting from the earliest phases of human development. The primary objective of this study is to identify pregnant women who may benefit from interventions designed to counteract metabolically induced inflammation and optimize lipid profiles.

## 2. Materials and Methods

### 2.1. Legal and Ethical Considerations

The current study was designed as a single-center study at the Obstetrics and Gynecology Clinic of the Timisoara Municipal Emergency Hospital, affiliated with the Victor Babes University of Medicine and Pharmacy from Timisoara, Romania, during the period January 2023–January 2024. This observational study secured ethical approval from the Institutional Review Board at The Obstetrics and Gynecology Clinic of the Timisoara Municipal Emergency Hospital, adhering to the principles set forth in the Declaration of Helsinki. Additionally, this study complies with the EU Good Clinical Practice Directive (2005/28/EC) and the guidelines provided by the International Council for Harmonization of Technical Requirements for Pharmaceuticals for Human Use (ICH), which emphasize informed consent, scientific validity, and the safeguarding of participants’ health and rights.

In alignment with the General Data Protection Regulation (GDPR) and relevant national data protection laws, our study incorporates stringent measures to protect personal data. All patient information is anonymized before analysis, effectively removing any identifiers that could be traced back to individuals. All patients included in the study signed an informed consent for data acquisition, dissemination, and publication of research studies.

### 2.2. Inclusion Criteria and Study Groups

Participants eligible for inclusion in this study were categorized into two groups. Group A comprised pregnant women aged between 15 and 40 years, who had no pathological conditions associated with pregnancy, maintained a normal lipid profile, and demonstrated a low risk of developing gestational diabetes according to the Fetal Medicine Foundation’s criteria at the first trimester screening. These criteria included age, body mass index, racial origin, conception method, family history of diabetes, parity, and a fasting glucose level below 92 mg/dL during the first trimester. Group B included pregnant women within the same age range who presented with dyslipidemia, characterized by triglycerides exceeding 150 mg/dL and HDL cholesterol levels below 60 mg/dL, alongside a high risk of developing gestational diabetes as identified by the same criteria used for Group A but with a fasting glucose level above 92 mg/dL in the first trimester. The time of inclusion in the study was at the first antenatal visit, between 8 and 14 weeks of gestation.

Exclusion criteria for the study were any fetus diagnosed with congenital malformations or chromosomal diseases during prenatal screenings. Pregnant women with severe systemic diseases unrelated to pregnancy, such as chronic kidney disease or heart failure, which could interfere with study outcomes, were also excluded. Additionally, women with pre-existing type 1 or type 2 diabetes or known cardiovascular diseases prior to pregnancy were not eligible for inclusion.

### 2.3. Study Variables

For this study, we selected and gathered a set of variables to assess the impact of maternal risk factors on fetal intima–media thickness of the abdominal aorta. The variables collected included demographic information such as the number of gestations and parity, as well as maternal age and gestational age. Clinical data encompassed diet adherence, insulin usage, type of delivery (cesarean section or natural birth), and the presence of conditions like preeclampsia and hypertensive disorders specific to pregnancy. Additionally, we evaluated fetal health indicators such as Apgar scores and fetal weight at birth. Biochemical and physiological measurements included maternal lipid profiles (HDL, LDL, triglycerides), systemic inflammation markers (tumor necrosis factor-alpha, hypersensitive C-reactive protein), and body mass index at the time of ultrasound examination. Crucially, the primary outcome measure, the intima–media thickness of the fetal abdominal aorta, was recorded at 28 weeks of gestation to provide a detailed analysis of cardiovascular risk potential from an early age. Finally, the compound scores were calculated and compared. Compound score 1 was calculated based on the lipid profile as [(BMI × LDL × Triglycerides)/(HDL × 1000)], while compound score 2 was calculated as log(TNF-alpha + 1) × hsCRP.

### 2.4. Definitions

**Figure 1:**
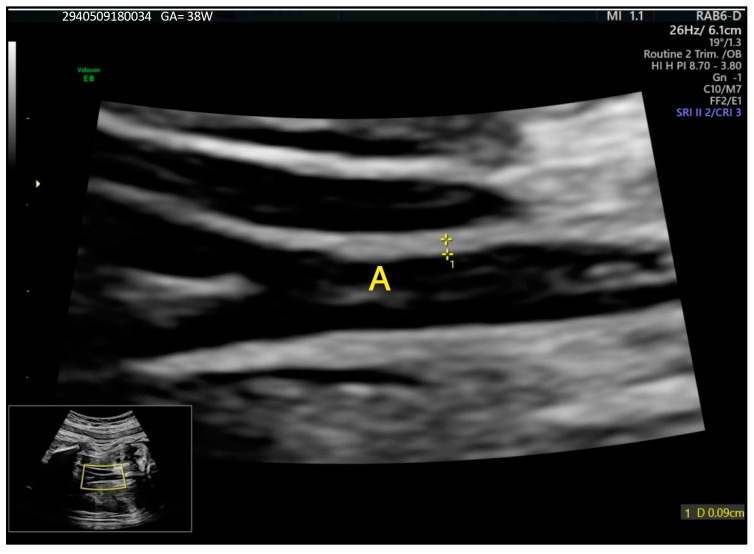
IMT of fetal abdominal aorta at 28 weeks of gestation. A—aorta. The intima is the echoic zone adjacent to the lumen and the media is the hypoechoic zone just outside the intima.

This study measured fetal IMT as a potential indicator of prenatal cardiovascular health to assess potential indicators of increased cardiovascular risk. In this study, the fetal abdominal IMT percentiles relative to gestational age were determined using the nomogram established by previous studies [10], which was specifically designed for fetal measurements. However, to date, there is no consensus or guideline for this type of measurement; therefore, the values of 0.9–1.1 mm were used in this study for the 75th and 90th percentiles. Therefore, IMT was defined as follows: values below the 75th percentile were considered normal, those between the 75th and 90th percentile were viewed as borderline, and values at or above the 90th percentile indicated an increased risk of atherosclerotic disease development. Measurements were performed as in the below example (Figure 1).

Gestational hypertension was defined as blood pressure consistently at or above 140/90 mmHg before the 20th week of gestation, and gestational hypertension, occurring after the 20th week without proteinuria. According to internal hospital criteria where the study took place, preeclampsia was characterized by blood pressure exceeding 140/90 mmHg and significant proteinuria after 20 weeks in a previously normotensive woman.

In this study, we adopted the universal screening approach recommended by the American College of Obstetricians and Gynecologists (ACOG) for detecting gestational diabetes. Initially, all pregnant women underwent a Glucose Challenge Test (GCT) between 24 and 28 weeks of gestation, which did not require fasting. If the GCT results were elevated, a follow-up Oral Glucose Tolerance Test (OGTT) was performed, requiring overnight fasting and multiple blood glucose readings post-glucose consumption to confirm the diagnosis. This two-step method ensured comprehensive screening and management of gestational diabetes.

### 2.5. Statistical Analysis

Data handling and statistical evaluations were conducted using SPSS Statistics version 25.0. Continuous data were represented as mean values ± standard deviation (SD), and categorical data were expressed in terms of frequencies and percentages. For the analysis of continuous variables among different groups, the Mann–Whitney U test was utilized due to the non-normal distribution of the clinical score data, while the chi-square test was employed for categorical variables. Receiver operating characteristic (ROC) curves were generated to assess the predictive accuracy of the clinical scores, with the calculation of the area under the curve (AUC) and the determination of sensitivity and specificity values. The “best cutoff” values were determined based on the highest statistical significance achieved between sensitivity and specificity for each parameter. Multiple logistic regression was applied to ascertain the odds ratios for atherosclerosis disease development, with a *p*-value of less than 0.05 indicating statistical significance. A two-sided *p*-value was employed in the current study.

## 3. Results

This study compared maternal risk factors and outcomes for the fetal abdominal aorta intima–media thickness (IMT) measured at 28 weeks of gestation, stratified by whether the IMT was below or above the 75th percentile. Notably, the maternal body mass index (BMI) was significantly higher in the group with an IMT above the 75th percentile (27.5 ± 4.1) compared to those below (24.7 ± 3.6), with a *p*-value of less than 0.001. Similarly, significant differences were observed in the birth weight, with the babies in the higher IMT group weighing an average of 4069 ± 526 g compared to 3166 ± 473 g in the lower IMT group (*p* < 0.001). The prevalence of preeclampsia and gestational hypertension was also notably higher in the group above the 75th percentile, recorded at 46.3% and 36.6%, respectively, compared to 27.9% and 17.1% in the group below the 75th percentile, with respective *p*-values of 0.031 and 0.010.

Additionally, the analysis showed a slight increase in the gestational age at birth in the group above the 75th percentile (38.2 ± 1.2 weeks) compared to the group below the 75th percentile (37.5 ± 2.0 weeks), with a statistically significant difference (*p* = 0.045). The differences in the APGAR scores at birth were also statistically significant; a lower proportion of newborns in the higher IMT group scored between 9 and 10 compared to those in the lower IMT group, with 31.7% versus 53.2%, respectively (*p* = 0.005), as seen in Table 1.

The high-density lipoprotein (HDL) levels were notably lower in the group with an IMT above the 75th percentile, averaging 59.12 ± 10.09 mg/dL, compared to 68.43 ± 8.16 mg/dL in the lower IMT group, with a statistically significant difference (*p* < 0.001). Likewise, the low-density lipoprotein (LDL) levels were higher in the group above the 75th percentile at 101.93 ± 19.51 mg/dL versus 94.75 ± 17.76 mg/dL in the group below the 75th percentile, also showing statistical significance (*p* = 0.032).

Furthermore, the inflammatory markers displayed significant elevations correlating with higher IMT measurements. The tumor necrosis factor-alpha (TNF-alpha) levels were considerably higher in the group with IMT measurements above the 75th percentile, averaging 8.66 ± 3.87 pg/mL compared to 4.96 ± 3.37 pg/mL in the lower IMT group (*p* < 0.001). The high-sensitivity C-reactive protein (hsCRP) levels followed a similar pattern, with values of 0.94 ± 0.46 mg/L in the higher IMT group versus 0.60 ± 0.52 mg/L in the lower IMT group, further indicating a significant association (*p* < 0.001), as seen in Table 2.

The TNF-alpha values had a cutoff value of 10.66 pg/mL, achieving a sensitivity of 81.63% and specificity of 70.59%, with an area under the curve (AUC) of 0.682 and a statistically significant *p*-value of less than 0.0001. Similarly, the hsCRP demonstrated a high diagnostic accuracy with a cutoff value of 2.92 mg/L, yielding a sensitivity of 84.41% and specificity of 79.01%, and the highest AUC of 0.738 among all the tested parameters, also with a *p*-value of less than 0.0001.

The lipid parameters such as HDL, LDL, and triglycerides presented lower diagnostic utility. The cutoff for HDL was set at 44.61 mg/dL with a sensitivity of 43.36% and specificity of 58.40%, accompanied by a low AUC of 0.403, indicating a poor predictive value (*p*-value of 0.825). The LDL and triglycerides similarly showed insufficient predictive powers with AUCs of 0.412 and 0.506, respectively, and non-significant *p*-values. Moreover, compound score 2 had an excellent cutoff value of 6.18, resulting in a high sensitivity of 86.35% and specificity of 81.42%, with an AUC of 0.789 and a significant *p*-value of less than 0.0001, as presented in Table 3.

Tumor necrosis factor-alpha (TNF-alpha) and high-sensitivity C-reactive protein (hsCRP) were strongly associated with an increased risk of fetal atherosclerotic disease, with hazard ratios (HRs) of 2.21 (95% CI: 1.15–5.28) and 2.87 (95% CI: 1.11–4.23), respectively, both achieving statistical significance with *p*-values of less than 0.0001. Similarly, the compound scores developed for the study, designed to integrate multiple risk factors, showed a notably increased risk with HRs of 4.05 (95% CI: 1.09–6.21) for compound score 1 and 4.27 (95% CI: 1.19–8.32) for compound score 2, also with *p*-values of less than 0.0001.

Conversely, other traditional lipid markers such as HDL, LDL, and triglycerides did not show statistically significant associations with the development of fetal atherosclerotic disease. The hazard ratios for HDL and LDL were 0.93 (95% CI: 0.88–1.36) and 1.06 (95% CI: 1.01–1.12), respectively, as presented in Table 4.

## 4. Discussion

### 4.1. Analysis of Findings

The findings of this study underscore the significant role of inflammatory markers in predicting the fetal intima–media thickness and, by extension, potential atherosclerotic risks during gestation. The robust associations found with TNF-alpha and hsCRP in both the cutoff analysis and the regression analysis all point to a critical link between maternal inflammation and fetal vascular health. Specifically, the cutoff values for TNF-alpha and hsCRP indicated high sensitivity and specificity, suggesting that elevated levels of these markers can reliably predict a higher IMT, a surrogate marker for atherosclerosis.

Moreover, the compound scores integrating multiple risk factors demonstrated an even greater predictive power, suggesting that a multi-marker approach may enhance the accuracy of prenatal screening for fetal cardiovascular risks. The compound scores, particularly compound score 2, which incorporates the hsCRP and the TNF-alpha values, showed the highest AUC and hazard ratios, indicating an excellent capability to identify fetuses at high risk of atherosclerotic disease development.

However, the study also highlighted the limited predictive utility of traditional lipid markers (HDL, LDL, and triglycerides) for fetal atherosclerotic disease development. Despite established roles in cardiovascular risk assessment in adults, these markers did not exhibit significant associations in the prenatal context, as shown by their relatively low hazard ratios and non-significant *p*-values. This discrepancy might be due to physiological changes in lipid metabolism during pregnancy or the timing of measurement and suggests that lipid profiles may not be reliable indicators of the fetal cardiovascular risk. The prominent role of inflammation-related markers over traditional lipid markers in this study could shift the focus towards anti-inflammatory strategies in managing maternal–fetal health, potentially opening new avenues for preventive measures against early-onset cardiovascular disease.

Other studies such as Gomez-Roig et al. [21] and Cosmi et al. [22] identified significant alterations in the aortic intima–media thickness (aIMT) and diameter among intrauterine growth restriction (IUGR) fetuses compared to their counterparts. Gomez-Roig et al. reported that IUGR fetuses had the highest median aIMT of 0.504 mm, significantly higher than small for gestational age (SGA) fetuses at 0.466 mm and appropriate for gestational age (AGA) fetuses at 0.471 mm, with a *p*-value of 0.023. Similarly, Cosmi et al. found median aIMT values that were significantly higher in IUGR fetuses at 1.9 mm compared to AGA fetuses at 1.15 mm in utero (*p* < 0.001), persisting post-birth with an aIMT of 2.4 mm for IUGR and 1.03 mm for AGA. These findings highlight the association of IUGR with early vascular changes, suggesting a potential link to increased cardiovascular risk that begins in utero and may extend into early childhood.

In a similar manner, the study by Niemczyk et al. [11] focused on vascular adaptations during and after pregnancy, revealing that common carotid artery (CCA) intima–media thickness increased persistently from the second trimester (0.546 mm [SE 0.01]) and remained elevated even 2.7 years postpartum (0.581 mm [SE 0.02]), despite the inter-adventitial diameter (IAD) returning to pre-pregnancy levels. These findings suggest a long-term impact of pregnancy on vascular structures, potentially contributing to future cardiovascular disease (CVD) risk. Likewise, the study by Visentin et al. [23] examined the predictive role of the fetal aortic intima–media thickness (aIMT) for late-onset gestational hypertension, with the aIMT serving as a significant predictor in a multivariate model that also included maternal and fetal parameters. This model, which assessed the fetal aIMT at 29 to 32 weeks’ gestation, demonstrated a substantial predictive accuracy with an area under the curve of 81.07%.

Moreover, the study by Takeshi Shimizu et al. [24] highlighted the significance of the aortic intima–media thickness as an early marker for subclinical vasculopathy in preschool children born preterm, showing that these children had a significantly higher mean aortic IMT (577 μm) compared to their term-born counterparts (517 μm) with a *p*-value of 0.003. This finding suggests an early onset of vascular changes that may predispose these individuals to metabolic syndromes and cardiovascular diseases later in life. Similarly, the study conducted by Vylyny Chat et al. [25] in Bangladesh found a notable association between the number of children and the carotid intima–media thickness (cIMT) in women, where the cIMT increased by 4.5 μm for each additional child and was significantly higher in women with four or more children, indicating a potential role of repeated pregnancies in accelerating atherosclerotic disease.

Sarikabadayi et al. [26] explored the early signs of preclinical atherosclerosis in infants of diabetic mothers (IDMs), finding a significantly increased umbilical artery intima–media thickness (uIMT) and wall thickness (ruWT) in the large for gestational age (LGA) group of IDMs compared to both the appropriate for gestational age (AGA) group and a control group. Specifically, the LGA-IDM group displayed markedly higher uIMT and ruWT values, which correlated with the elevated levels of insulin, C-peptide, and insulin resistance measured by HOMA-IR (*p* < 0.001 for insulin and HOMA-IR, *p* = 0.018 for C-peptide). Similarly, the study by Koklu et al. [27] also highlighted the relationship between macrosomia, maternal diabetes, and early vascular changes, showing that macrosomic neonates of diabetic mothers had a significantly higher aortic intima–media thickness (0.56 ± 0.06 mm) compared to that of healthy neonates (0.39 ± 0.03 mm). Moreover, these infants exhibited altered lipid profiles, which are indicative of an enhanced atherogenic risk, potentially predisposing them to atherosclerosis later in life.

In a similar manner, the study by Tamer Gunes et al. [28] investigated the impact of maternal smoking on the neonatal aortic intima–media thickness (aIMT), revealing that neonates whose mothers smoked during pregnancy had a significantly higher aIMT (0.455 ± 0.009 mm) compared to controls (0.403 ± 0.029 mm), along with a reduction in birth weight and non-significant decreases in the serum IGF-I and IGFBP-3 levels. These findings suggest an early initiation of vascular changes that may contribute to atherosclerosis later in life. Correspondingly, the study by Esad Koklu et al. [29] on intrauterine growth-restricted (IUGR) neonates of healthy mothers also showed an increased aIMT (0.45 ± 0.03 mm in IUGR vs. 0.39 ± 0.04 mm in controls), with significantly lower levels of serum IGF-I and leptin, underscoring the role of compromised fetal growth and altered endocrine parameters in early vascular remodeling.

Predicting a higher fetal intima–media thickness through ultrasound at 28 weeks of gestation holds significant clinical utility. By identifying elevated IMT, clinicians can pinpoint fetuses at an increased risk of developing cardiovascular diseases later in life, allowing for the implementation of early intervention strategies. These may include targeted nutritional guidance, the optimized management of maternal health factors such as dyslipidemia and inflammation, and possibly pharmacological interventions if deemed safe and necessary. Such proactive measures could not only improve the immediate outcomes of pregnancy but also contribute to the long-term health of the child by potentially reducing the burden of cardiovascular diseases. Furthermore, this predictive capability enhances prenatal care by facilitating a more personalized approach, enabling healthcare providers to counsel and manage expectant mothers more effectively based on the identified risks.

### 4.2. Study Limitations

This study, while providing important insights, has several limitations that warrant consideration. First, its observational design restricts causality inference between the identified risk factors and an increased fetal IMT. Second, the study’s population was limited to a specific geographic area, potentially limiting the generalizability of the findings to diverse populations. Third, despite rigorous data collection, potential confounders such as detailed dietary intake, physical activity levels, and socioeconomic factors were not comprehensively accounted for, which could influence maternal inflammation markers and lipid profiles. Additionally, the measurement of the fetal IMT at only one gestational age point may not fully capture the dynamic changes throughout pregnancy. The reliance on specific biochemical markers and their thresholds may also not encompass all relevant aspects of the fetal cardiovascular risk, suggesting the need for a broader array of biomarkers in future studies.

## 5. Conclusions

This study demonstrates that significant associations between elevated maternal inflammation markers (TNF-alpha and hsCRP) and adverse pregnancy outcomes (such as higher birth weight and increased gestational hypertension) indicate a potentially significant increase in the fetal IMT, indicating a prenatal origin of potential cardiovascular risks. The strong correlations found with the compound scores, particularly compound score 2, that comprised both TNF-alpha and hsCRP values, suggest that integrating multiple maternal risk factors provides a more effective predictive tool for identifying fetuses at risk of developing atherosclerosis. Future research should focus on longitudinal designs, incorporate a broader range of biomarkers, and expand participant diversity to validate and extend these findings, ultimately enhancing prenatal care and cardiovascular risk stratification from gestation.

## Figures and Tables

**Table 1:** Characteristics of study participants based on intima–media thickness (IMT).

Variables	Below 75th % IMT (*n* = 111)	Above 75th % IMT (*n* = 41)	*p*-Value
Maternal age, years (mean ± SD)	31.4 ± 5.3	32.4 ± 5.1	0.288
Maternal BMI (mean ± SD)	24.7 ± 3.6	27.5 ± 4.1	<0.001
Number of gestations (gravidity)			0.067
1	40%	20%	
2	34%	46%	
>2	26%	34%	
Cesarean birth	95%	88%	0.151
Preeclampsia	28%	46%	0.031
Insulin necessity	14%	27%	0.075
Gestational hypertension	17%	37%	0.010
Diastolic dysfunction	11%	20%	0.158
Gestational age at birth, weeks (mean ± SD)	37.5 ± 2.0	38.2 ± 1.2	0.045
Birth weight (mean ± SD)	3166.5 ± 473.2	4068.78 ± 525.8	<0.001
APGAR score			0.005
9–10	53%	32%	
7–8	37%	39%	
<7	10%	29%	
IMT, (median [IQR])	0.721 [0.608–0.834]	0.954 [0.916–0.992]	<0.001

IMT—intima–media thickness; SD—standard deviation; BMI—body mass index; APGAR—appearance, pulse, grimace, activity and respiration.

**Table 2:** Lipid and inflammatory markers based on intima–media thickness (IMT).

Variables (Mean ± SD)	Below 75th % IMT (*n* = 111)	Above 75th % IMT (*n* = 41)	*p*-Value
HDL	68.43 ± 8.16	59.12 ± 10.09	<0.001
LDL	94.75 ± 17.76	101.93 ± 19.51	0.032
Triglycerides	128.34 ± 24.31	130.63 ± 22.32	0.599
TNF-alpha	4.96 ± 3.37	8.66 ± 3.87	<0.001
hsCRP	0.60 ± 0.52	0.94 ± 0.46	<0.001

SD—standard deviation; IMT—intima–media thickness; HDL—high-density lipoprotein; LDL—low-density lipoprotein; TNF—tumor necrosis factor; hsCRP—High-Sensitivity C-reactive protein.

**Table 3:** Best cutoff values for intima–media thickness suggestive of increased cardiovascular risk.

Parameters	Best Cutoff Value	Sensitivity	Specificity	AUC	*p*-Value
BMI	31.06	65.49	57.34	0.461	0.344
HDL	44.61	43.36	58.40	0.403	0.825
LDL	108.25	56.12	59.56	0.412	0.517
Triglycerides	149.10	57.98	51.67	0.506	0.394
TNF-alpha	10.66	81.63	70.59	0.682	<0.0001
hsCRP	2.92	84.41	79.01	0.738	<0.0001
Compound score 1	11.27	72.08	66.97	0.622	0.008
Compound score 2	6.18	86.35	81.42	0.789	<0.0001

HDL—high-density lipoprotein; LDL—low-density lipoprotein; TNF—tumor necrosis factor; hsCRP—High-Sensitivity C-reactive protein; AUC—area under curve.

**Table 4:** Regression analysis for increased cardiovascular risk based on IMT.

Factors Above the Best Cutoff	Hazard Ratio	95% CI	*p*-Value
BMI	1.09	(1.03–1.15)	0.102
HDL	0.93	(0.88–1.36)	0.078
LDL	1.06	(1.01–1.12)	0.314
Triglycerides	1.04	(0.99–1.89)	0.129
TNF-alpha	2.21	(1.15–5.28)	<0.0001
hsCRP	2.87	(1.11–4.23)	<0.0001
Compound score 1	4.05	(1.09–6.21)	<0.0001
Compound score 2	4.27	(1.19–8.32)	<0.0001

HDL—high-density lipoprotein; LDL—low-density lipoprotein; TNF—tumor necrosis factor; hsCRP—High-Sensitivity C-reactive protein; AUC—area under curve.

## Data Availability

The data presented in this study are available on request from the corresponding author.
